# Supplementary material for: Heterogeneous plasticity of amygdala interneurons in associative learning and extinction
Source: Nat Commun. 2025 Nov 11;16:9926. doi: 10.1038/s41467-025-66122-y (PMC12614800; doi:10.1038/s41467-025-66122-y)
Supplement: Supplementary file 1 — Supplementary Information [file 41467_2025_66122_MOESM1_ESM.pdf]

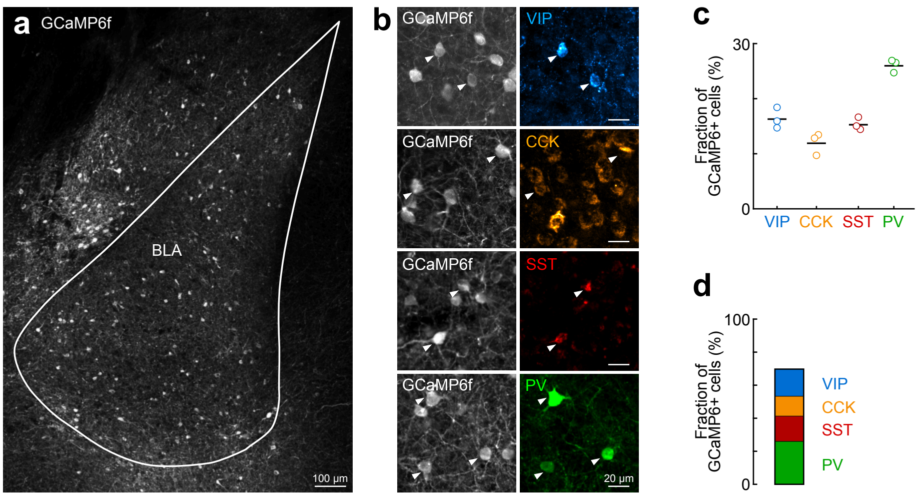

Supplementary Figure 1: Expression of GCaMP6 in *GAD2-Cre* mice

a, Representative example of GCaMP6 expression in the amygdala after injection of AAV2/9.CAG.flex.GCaMP6f in *GAD2-Cre* mice (N = 3). Maximum intensity projection; outline delineates the BLA. **b**, Examples of GCaMP6 co-expression with main BLA interneuron marker genes from the same mouse (VIP, vasoactive intestinal peptide; CCK, cholecystokinin; SST, somatostatin; PV, parvalbumin). Arrowheads point to GCaMP6+ neurons immunopositive for the respective marker. Note that the peptide CCK is not exclusively expressed in interneurons but also in excitatory BLA projection neurons[75]. **c**, Fraction of GCaMP6+ neurons immunopositive for VIP, CCK, SST and PV in the BLA after AAV injection in *GAD2-Cre* mice. PV and SST expression were analysed in the same sections (N = 3 mice, n = 541 ± 53 GCaMP+ cells), and CCK and VIP together in adjacent sections of the same mice (n = 624 ± 97 cells). Dots are individual animals; line indicates the mean. **d**, Combined fraction of GCaMP6+ neurons immunopositive for VIP, CCK, SST or PV (mean of N = 3 mice).

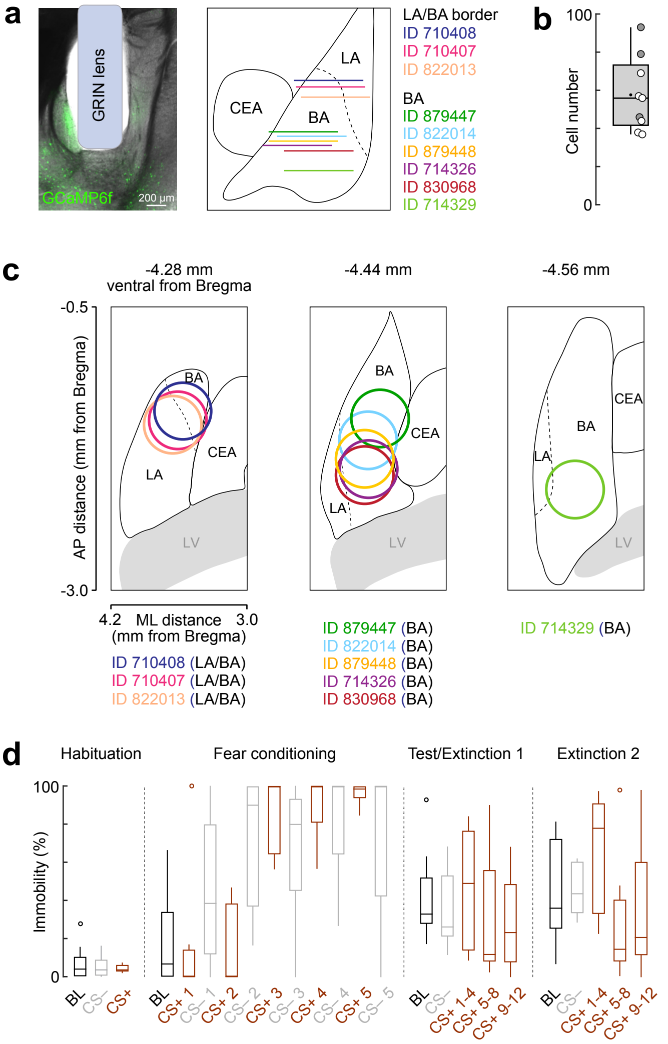

Supplementary Figure 2: Imaging of basolateral amygdala interneurons during fear and extinction learning

a, Representative implant site (ID 879448) and schematic illustrating all reconstructed implant sites of GRIN lenses (lens front) within the BLA of *GAD2-Cre* mice for deep brain imaging experiments matched to a mouse brain atlas (N = 9 mice). LA, lateral amygdala; BA, basal amygdala; CEA, central amygdala. **b**, Average cell numbers recorded across the four-day paradigm (N = 9). Tukey box-and-whisker plot illustrates median values, 25th and 75th percentiles, and min to max whiskers, dot indicates the mean. Circles represent individual animals (open circles, imaging sites in the basal amygdala (N = 6); filled circles, at the border of the lateral and basal amygdala (N = 3). **c**, Schematic illustrating all reconstructed implant sites of GRIN lenses (lens front) on horizontal mouse brain atlas planes (N = 9 mice). LA, lateral amygdala; BA, basal amygdala; CEA, central amygdala; LV, lateral ventricle. **d**, Immobility levels throughout the fear conditioning and extinction paradigm in GRIN lens-implanted *GAD2-Cre* mice (N = 9). Tukey box-and-whisker plots illustrates median values, 25th and 75th percentiles, and min to max whiskers, circles indicate outliers.

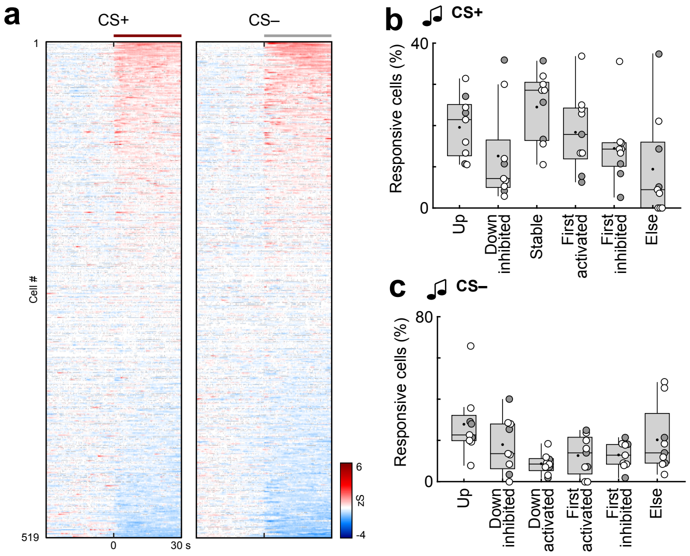

Supplementary Figure 3: CS coding in amygdala interneurons during fear conditioning

a, Heatmap of CS+ and CS– responses in BLA interneurons during conditioning, averaged across all five trials and ordered individually by response amplitude (n = 519 cells from N = 9 mice). Lines indicate CS duration. **b**, Fraction of interneurons according to CS+ cluster membership across animals (see Figure 4; N = 9). Friedman test ($\chi2$ = 11.26), *p* = 0.0465; followed by Dunn's multiple comparisons (non-significant). **c**, Fraction of interneurons according to CS– cluster membership across animals (see Figure 4; N = 9).

Tukey box-and-whisker plots in b and c show median values, 25th and 75th percentiles, and min to max whiskers with exception of outliers, dots indicate the mean. Circles represent individual animals (open circles, imaging sites in the basal amygdala (N = 6); filled circles, at the border of the lateral and basal amygdala (N = 3). Additional details of statistical analyses are provided in Supplementary Table 1.

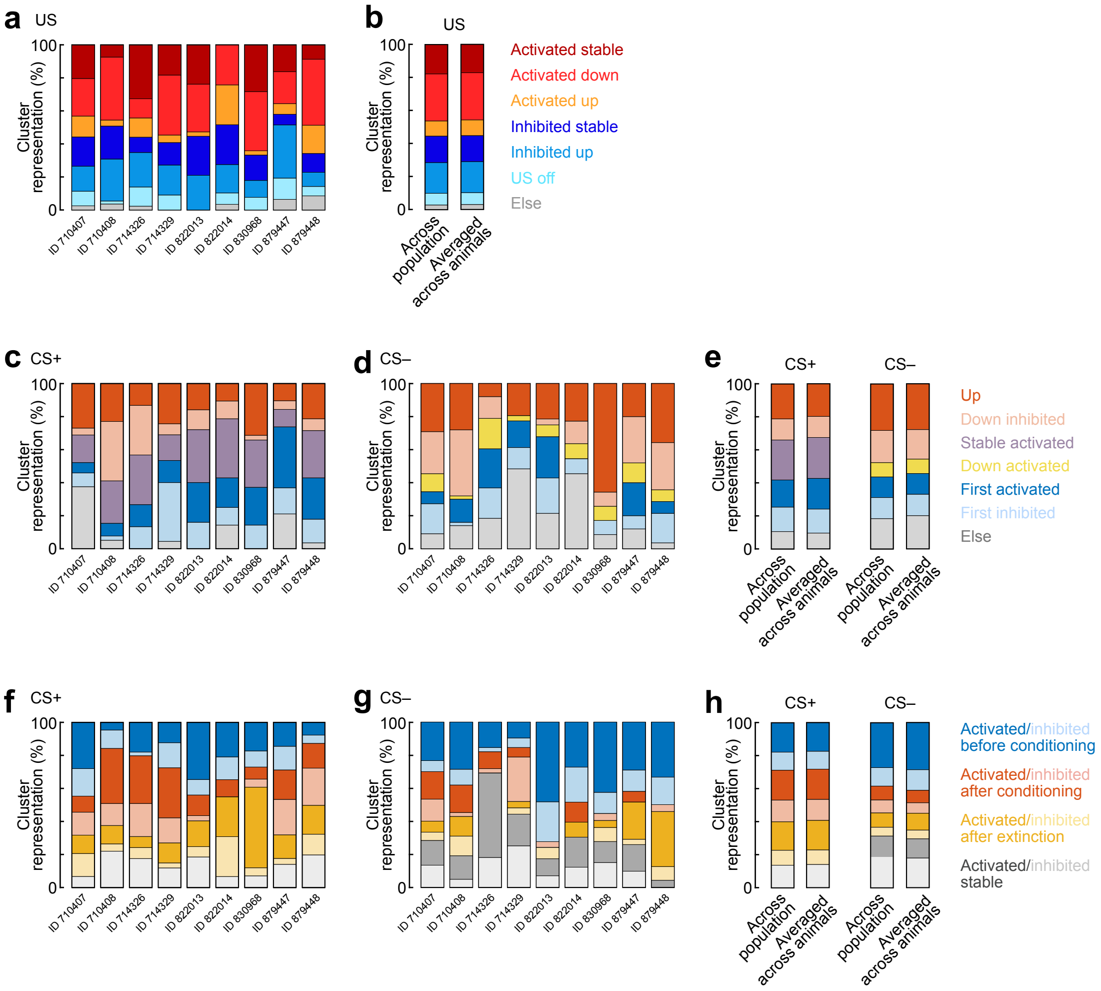

Supplementary Figure 4: Variability of interneuron response types across animals

a, Proportion of cells in US clusters plotted for all individual *GAD2-Cre* mice (N = 9). **b**, Comparison of "across population" analysis (n = 393 cells, as shown in Figure 3g for direct comparison) with results averaged across animals (N = 9). **c-d**, Proportion of cells in CS+ and CS– clusters during fear conditioning plotted for all individual GAD2-Cre mice (N = 9). **e**, Comparison of "across population" analysis (CS+, n = 297 cells; CS–, n = 312; as shown in Figure 4h) with results averaged across animals (N = 9). **f-g**, Proportion of cells in across-day plasticity clusters (CS+ and CS–) plotted for all individual *GAD2-Cre* mice (N = 9). **h**, Comparison of "across population" analysis (CS+, n = 365 cells; CS–, n = 357; as shown in Figure 5h) with results averaged across animals (N = 9).

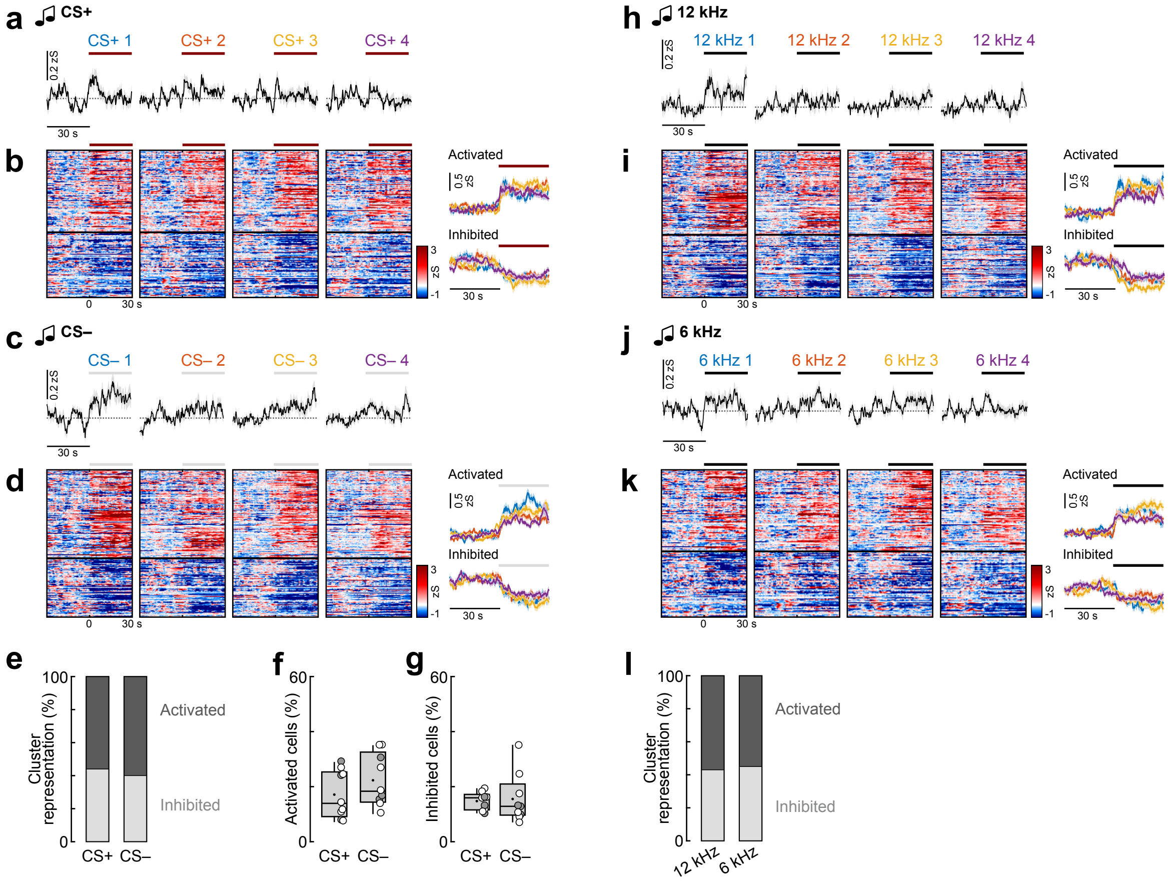

Supplementary Figure 5: Interneuron responses to auditory stimuli

a, Average traces of basolateral amygdala interneurons during the first four CS+ presentations during habituation (n = 519 cells from N = 9 mice; counterbalanced for 6 kHz and 12 kHz). Line indicates CS duration. **b**, Heatmap (left) of CS+ responses clustered into groups depending on their response pattern across the four presentations and corresponding average traces of clusters (right). n = 165 responsive cells; 'Activated', n = 92; 'Inhibited', n = 73. **c**, Average traces of basolateral amygdala interneurons during the first four CS– presentations during habituation (n = 519; counterbalanced for 6 kHz and 12 kHz). **d**, Heatmap (left) of CS– responses clustered into groups depending on their response pattern across the four presentations and corresponding average traces of clusters (right). n = 185 responsive cells; 'Activated', n = 111; 'Inhibited', n = 74. **e**, Proportion of cells in CS+ and CS– clusters (CS+, n = 165; CS–, n = 185). **f**, Proportion of CS+ and CS– activated neurons during habituation (N = 9 mice). **g**, Proportion of CS+ and CS– inhibited neurons during habituation (N = 9 mice). **h**, Average traces of basolateral amygdala interneurons during the first four 12 kHz presentations during habituation (n = 519; later assigned to be CS+ or CS–). **i**, Heatmap (left) of 12 kHz responses clustered into groups depending on their response pattern across the four presentations and corresponding average traces of clusters (right). n = 202 responsive cells; 'Activated', n = 116; 'Inhibited', n = 86. **j**, Average traces of basolateral amygdala interneurons during the first four 6 kHz presentations during habituation (n = 519; later assigned to be CS+ or CS–). **k**, Heatmap (left) of 6 kHz responses clustered into groups depending on their response pattern across the four presentations and corresponding average traces of clusters (right). n = 148 responsive cells; 'Activated', n = 82; 'Inhibited', n = 66. **l**, Proportion of cells in 12 kHz and 6 kHz clusters (12 kHz, n = 202; 6 kHz, n = 148).

Average traces across panels are mean with s.e.m., Tukey box-and-whisker plots in f and g show median values, 25th and 75th percentiles, and min to max whiskers with exception of outliers, dots indicate the mean. Circles represent individual animals (open circles, imaging sites in the basal amygdala (N = 6); filled circles, at the border of the lateral and basal amygdala (N = 3). Additional details of statistical analyses are provided in Supplementary Table 1.

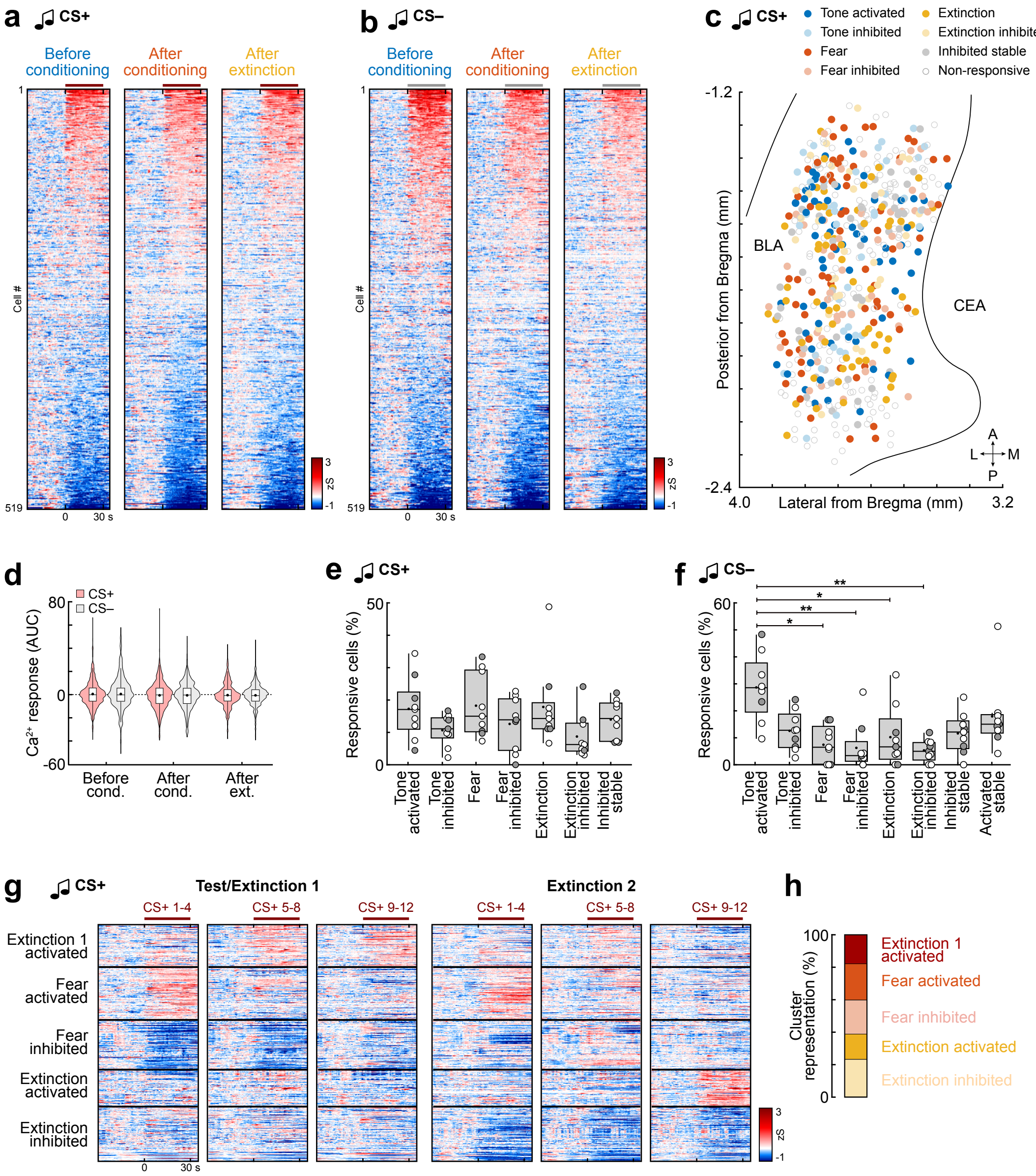

Supplementary Figure 6: CS encoding across learning days in basolateral amygdala interneurons

a, Heatmap of CS+ and **b**, CS– responses in BLA interneurons before conditioning, after conditioning and after extinction, averaged across the four CS presentations used for clustering (n = 519 cells from N = 9 mice). Line indicates CS duration. **c**, Location of interneurons (centroids) on the horizontal atlas plane colour-coded for CS+ response type across days (n = 519 cells from N = 9 mice). For easier assessment, different ventral planes of lens fronts (see Supplementary Figure 2) were combined on the same plane. Lines indicate BLA borders. **d**, Comparison of area under the curve (AUC) for CS+ and CS– responses in BLA interneurons (n = 519). **e**, Fraction of interneurons according to CS+ cluster membership across animals (see Figure 5; N = 9). **f**, Fraction of interneurons according to CS– cluster membership across animals (see Figure 5; N = 9). Friedman test ($\chi 2$ = 25.22), *p* = 0.0007, followed by Dunn's multiple comparisons (‘Tone activated’/activated before conditioning vs. ‘Fear’/activated after conditioning, *p* = 0.0494; ‘Tone activated’/activated before conditioning vs. ‘Fear inhibited’/inhibited after conditioning, *p* = 0.0072; ‘Tone activated’/activated before conditioning vs. ‘Extinction’/activated after extinction, *p* = 0.0212; ‘Tone activated’/activated before conditioning vs. ‘Extinction inhibited’/inhibited after extinction, *p* = 0.0022). **g**, Heatmap of CS+ responses in BLA interneurons clustered into groups depending on their response pattern across the two extinction sessions (n = 519; Extinction 1 activated, n = 93; Fear activated, n = 116; Fear inhibited, n = 109; Extinction activated, n = 81; Extinction inhibited, n = 120). **h**, Corresponding proportion of BLA interneurons in CS+ extinction clusters (n = 519).

Violin plots in d show distribution of all data points, Tukey box-and-whisker plots in d, e and f show median values, 25th and 75th percentiles, and min to max whiskers with exception of outliers, dots indicate the mean. Circles in e and f represent individual animals (open circles, imaging sites in the basal amygdala (N = 6); filled circles, at the border of the lateral and basal amygdala (N = 3). **p* < 0.05, ***p* < 0.01. Additional details of statistical analyses are provided in Supplementary Table 1.

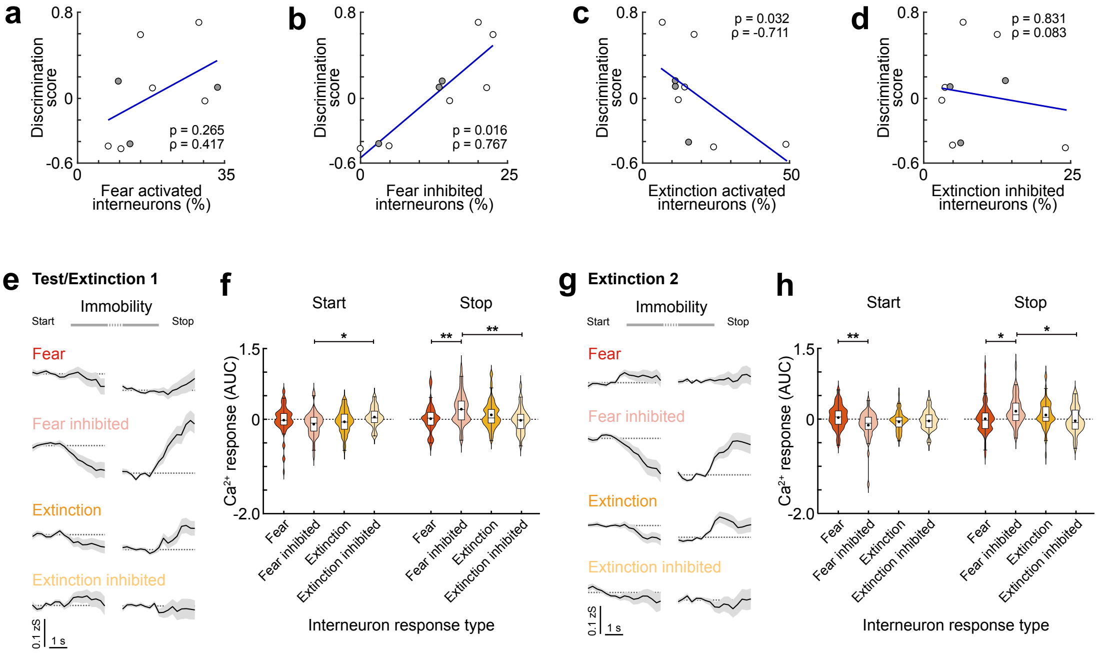

Supplementary Figure 7: Behavioural correlates of amygdala interneuron plasticity

a-d, Correlation of fractions of neurons in across-day CS+ plasticity clusters with learning-related performance in individual animals (N = 9) for **a**, Fear interneurons; **b**, Fear inhibited interneurons; **c**, Extinction interneurons; and **d**, Extinction inhibited interneurons. Lines indicate linear regression fit (shown for visualisation); correlation coefficient ρ reflects Spearman's rank-order correlation. **e**, Average traces of across-day CS+ plasticity clusters during immobility in Test/Extinction 1 session (see Figure 5; 'Fear'/activated after conditioning, n = 66; 'Fear inhibited'/inhibited after conditioning, n = 48; 'Extinction'/activated after extinction, n = 63; 'Extinction inhibited'/ inhibited after extinction, n = 33). **f**, Corresponding area under the curve (AUC) for immobility start and stop (2 s duration). Start: Kruskal Wallis test (H = 9.836), p = 0.0200 with *post hoc* comparison (Fear inhibited vs. Extinction inhibited, p = 0.0255). Stop: Kruskal Wallis test (H = 14.83), p = 0.0020 with *post hoc* comparison (Fear vs. Fear inhibited, p = 0.0057; Fear inhibited vs. Extinction inhibited, p = 0.0058). **g**, Average traces of the same across-day CS+ plasticity clusters during immobility in Extinction 2 session. **h**, Corresponding area under the curve (AUC) for immobility start and stop (2 s duration). Start: Kruskal Wallis test (H = 10.89), p = 0.0123 with *post hoc* comparison (Fear vs. Fear inhibited, p = 0.0095). Stop: Kruskal Wallis test (H = 14.19), p = 0.0027 with *post hoc* comparison (Fear vs. Fear inhibited, p = 0.0135; Fear inhibited vs. Extinction inhibited, p = 0.0132).

Average traces in e and g are mean with s.e.m., violin plots in f and h show distribution of all data points, Tukey box-and-whisker plots show median values, 25th and 75th percentiles, and min to max whiskers with exception of outliers, dots indicate the mean. Circles in a-d represent individual animals (open circles, imaging sites in the basal amygdala (N = 6); filled circles, at the border of the lateral and basal amygdala (N = 3). *p < 0.05, **p < 0.01. Additional details of statistical analyses are provided in Supplementary Table 1.

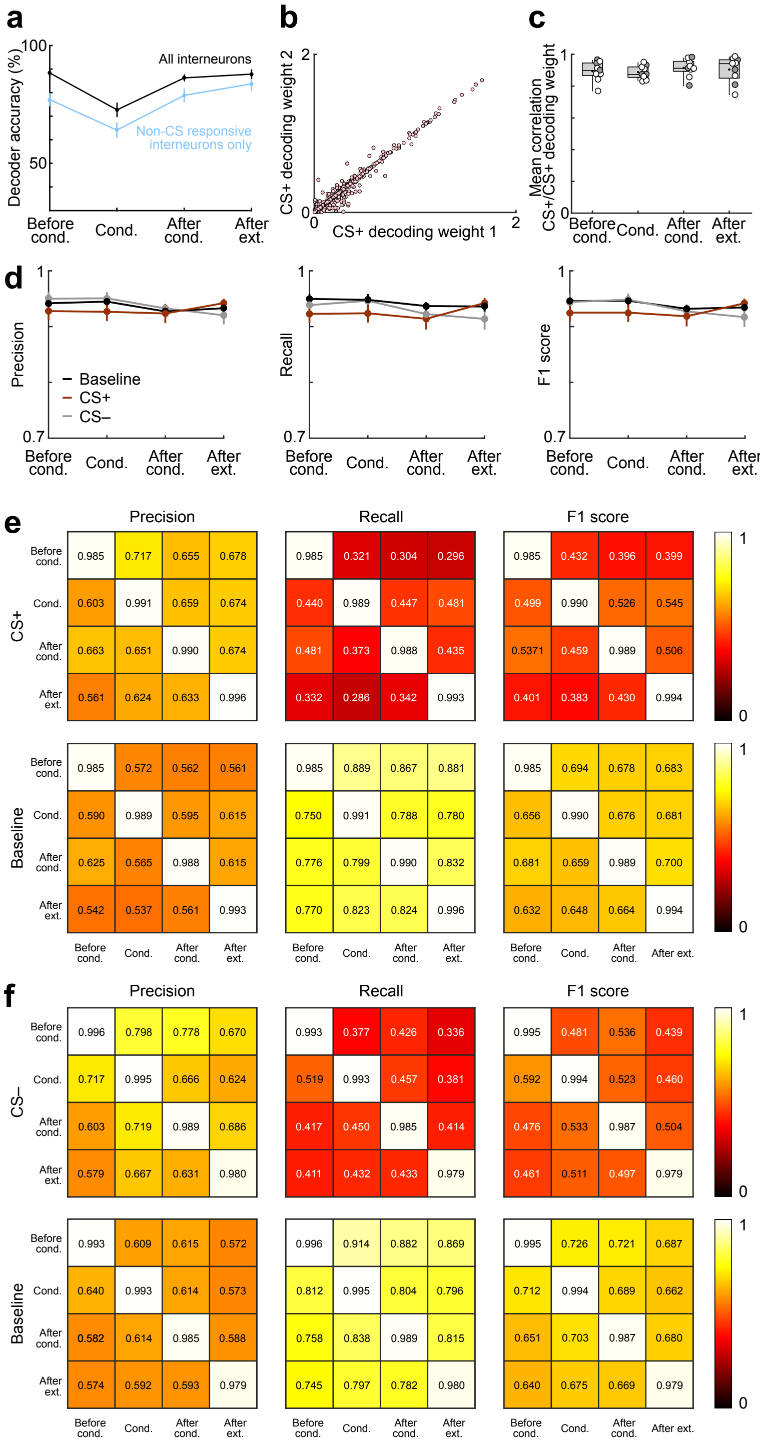

Supplementary Figure 8: Performance metrics of classifiers

a, Comparison of mean accuracy of multiclass intra-day decoders of CS+, CS– and baseline for each day of the behavioural paradigm, averaged across all animals (N = 9 mice) and iterations (n = 100 iterations). Light blue bars indicate the accuracy of decoders trained on a random sample of non-CS-responsive cells. Black bars represent the accuracy of control decoders trained on a random sample of cells selected from all interneurons (regardless of CS responsiveness), with the number of cells matched to those used in the non-CS decoders. **b**, Example scatterplot showing the absolute value of the decoding weight for the CS+ at iteration *i*, and at iteration *i+1*, from all animals and interneurons after conditioning (n = 519 cells, N = 9 mice). **c**, Average correlation between CS+$_i$ and CS+$_{i+1}$ decoding weights for each session, calculated per animal (N = 9 mice). Correlations were calculated between the decoding weights of the *ith* and the *ith+1* iteration, using a total of 200 iterations to obtain 100 correlation values. **d,** Precision, recall and F1 score calculated for each class independently on each day for the intraday multiclass decoders classifying CS+, CS– and baseline. **e,** Precision, recall and F1 score calculated for each class for the intra and inter day performance of the two-way decoder classifying CS+ and baseline. **f,** Precision, recall and F1 score calculated for each class for the intra and inter day performance of the two-way decoder classifying CS– and baseline.

Tukey box-and-whisker plots in c show median values, 25th and 75th percentiles, and min to max whiskers with exception of outliers, dots indicate the mean, circles represent individual animals (open circles, imaging sites in the basal amygdala (N = 6); filled circles, at the border of the lateral and basal amygdala (N = 3). All other plots display means from all animals (N = 9 mice) and all iterations (n = 100).

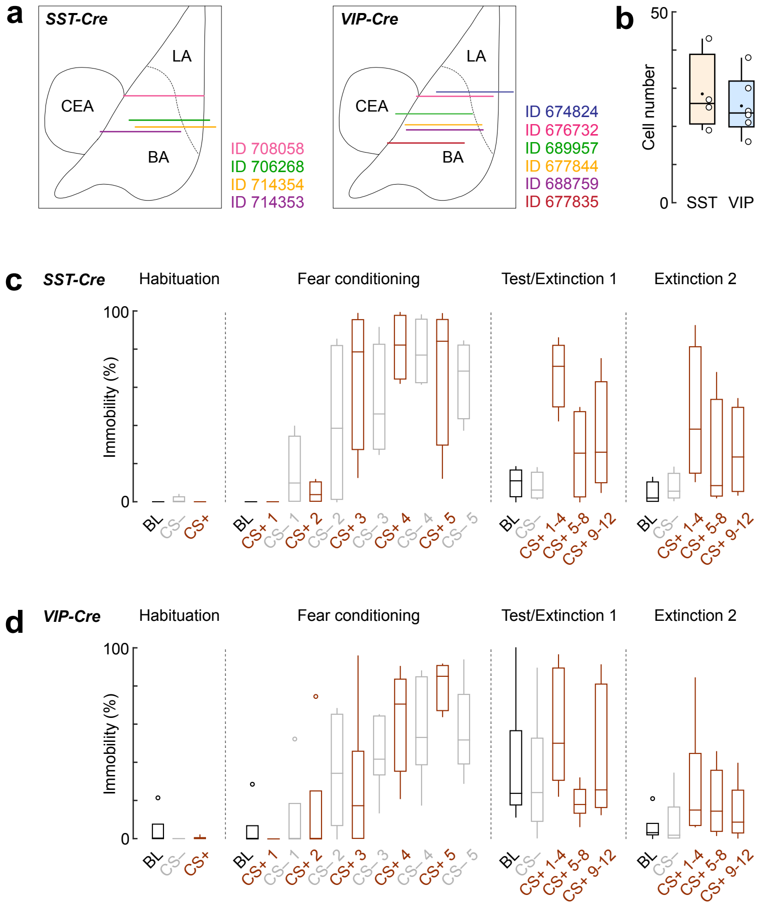

Supplementary Figure 9: Imaging of molecular interneuron subpopulations during fear and extinction learning

a, Schematic illustrating all reconstructed implant sites of GRIN lenses (lens front) within the BLA of *SST-Cre* (N = 4) and *VIP-Cre* mice (N = 6) for deep brain imaging experiments matched to a mouse brain atlas. LA, lateral amygdala; BA, basal amygdala; CEA, central amygdala. **b**, Average cell numbers recorded across the four-day paradigm (SST, N = 4; VIP, N = 6). Box-and-whisker plots show median values, 25th and 75th percentiles, and min to max whiskers, dots indicate the mean, circles are individual animals. **c**, Immobility levels throughout the fear conditioning and extinction paradigm in GRIN lens-implanted *SST-Cre* mice (N = 4). Tukey box-and-whisker plots illustrates median values, 25th and 75th percentiles, and min to max whiskers. **d**, Immobility levels throughout the fear conditioning and extinction paradigm in GRIN lens-implanted *VIP-Cre* mice (N = 6). Tukey box-and-whisker plots illustrate median values, 25th and 75th percentiles, and min to max whiskers, circles indicate outliers.

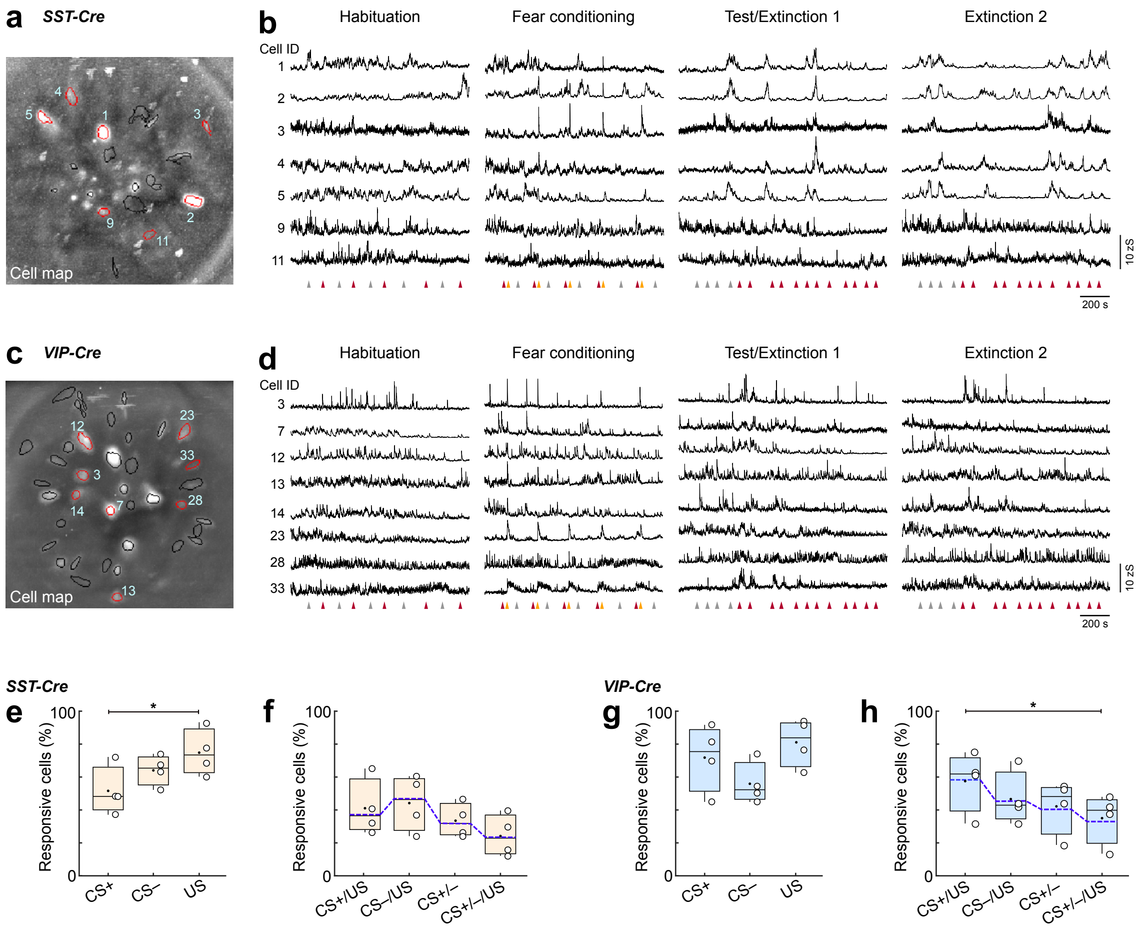

Supplementary Figure 10: Recording calcium activity across days in *SST-Cre* and *VIP-Cre* mice

a, Example field of view (maximum intensity projection across four-day paradigm) for an *SST-Cre* mouse. Circles indicate selected individual components. **b**, Representative example traces from the same animal. Cell IDs correspond to SST interneurons highlighted with red outlines in a. **c**, Example field of view (maximum intensity projection across four-day paradigm) for a *VIP-Cre* mouse. Circles indicate selected individual components. **d**, Representative example traces from the same animal. Cell IDs correspond to VIP interneurons highlighted with red outlines in c. **e**, Fraction of SST interneurons responsive to the CS+, CS– and US across distinct animals (N = 4). Friedman test ($\chi2 = 6.500$), $p = 0.0417$, followed by Dunn's multiple comparisons (CS+ vs. US, $p = 0.0400$). **f**, Proportion of overlap in CS+, CS– and US coding SST interneurons (N = 4). Blue line indicates chance overlap level. Friedman test ($\chi2 = 9.711$), $p = 0.0058$, followed by Dunn's multiple comparisons (non-significant). **g**, Fraction of VIP interneurons responsive to the CS+, CS– and US across distinct animals (N = 4). **h**, Proportion of overlap in CS+, CS– and US coding VIP interneurons (N = 4). Blue line indicates chance overlap level. Friedman test ($\chi2 = 8.595$), $p = 0.0168$, followed by Dunn's multiple comparisons (CS+/US vs. CS+/CS–/US, $p = 0.0370$).

Arrowheads in b and d indicate starting points of CS+ (red), CS– (grey) and US (yellow). Tukey box-and-whisker plots in e-h and f show median values, 25th and 75th percentiles, and min to max whiskers with exception of outliers, dots indicate the mean, circles represent individual animals. *$p < 0.05$. Additional details of statistical analyses are provided in Supplementary Table 1.

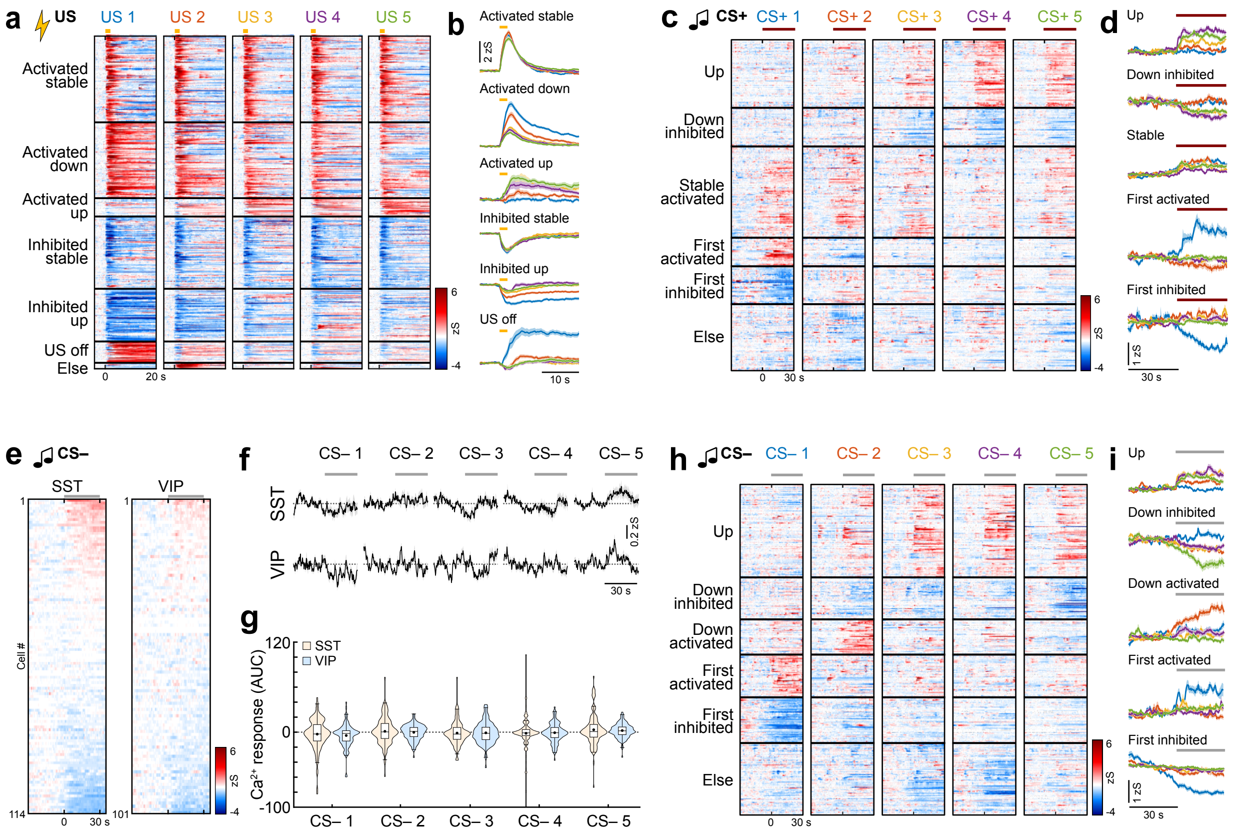

Supplementary Figure 11: Responses of interneuron subpopulations during fear learning

a, Heatmap of US responses in BLA interneurons (including data from all *GAD2-Cre*, *SST-Cre* and *VIP-Cre* mice) clustered into groups depending on their US response pattern across the five trials (n = 562 responsive cells; ‘Activated stable’, n = 146; ‘Activated down’, n = 128; ‘Activated up’, n = 31; ‘Inhibited stable’, n = 122; ‘Inhibited up’, n = 89; ‘US off’, n = 36; ‘Else’, n = 10). **b**, Average traces of US clusters shown in a. **c**, Heatmap of CS+ responses in BLA interneurons (including data from all *GAD2-Cre*, *SST-Cre* and *VIP-Cre* mice) clustered into groups depending on their response pattern across the five trials (n = 428 responsive cells; ‘Up’, n = 88; ‘Down inhibited’, n = 50; Stable activated’, n = 118; ‘First activated’, n = 37; ‘First inhibited’, n = 49; ‘Else’, n = 86). **d**, Average traces of CS+ clusters shown in c. **e**, Heatmap of SST and VIP BLA interneuron responses to the control CS– during conditioning (averaged across all five presentations), sorted by individual response amplitude (SST, n = 114 cells from N = 4 mice; VIP, n = 101, N = 4). **f**, Average CS– responses in SST and VIP interneurons across the five presentations (SST, n = 114; VIP, n = 101). **g**, Area under the curve (AUC) during CS– presentations in conditioning for SST and VIP interneurons (SST, n = 114; VIP, n = 101). **h**, Heatmap of CS– responses in BLA interneurons (including data from all *GAD2-Cre*, *SST-Cre* and *VIP-Cre* mice) clustered into groups depending on their response pattern across the five trials (n = 441 responsive cells; ‘Up’, n = 124; ‘Down inhibited’, n = 56; ‘Down activated’, n = 47; ‘First activated’, n = 57; ‘First inhibited’, n = 61; ‘Else’, n = 96). **i**, Average traces of CS– clusters shown in h.

Average traces in b, d, f and i are mean with s.e.m.; violin plots in g show distribution of all data points, Tukey box-and-whisker plots in g show median values, 25th and 75th percentiles, and min to max whiskers with exception of outliers, dots indicate the mean. Additional details of statistical analyses are provided in Supplementary Table 1.

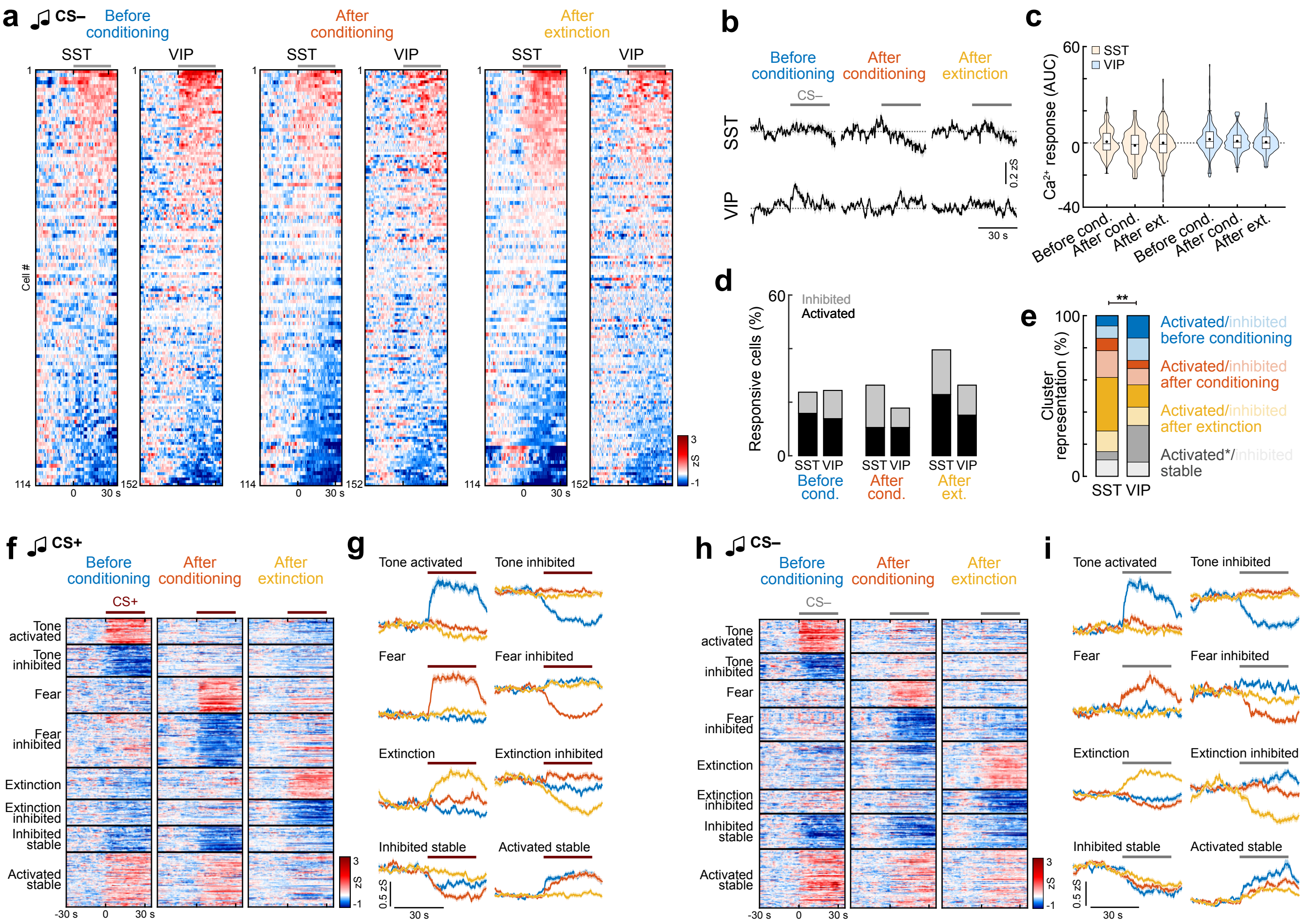

Supplementary Figure 12: Across-day plasticity of interneuron subpopulations

a, Heatmap of SST and VIP interneuron responses to the control CS– before conditioning, after conditioning and after extinction (averaged across four presentations each), sorted individually by response amplitude (SST, n = 114 cells from N = 4 mice; VIP, n = 152, N = 6). Grey line indicates CS duration. **b**, Corresponding average CS– responses in SST and VIP interneurons across days (SST, n = 114; VIP, n = 152). **c**, Area under the curve (AUC) during CS– presentations across learning for SST and VIP interneurons SST, n = 114; VIP, n = 152). **d**, Proportions of responsive neurons across the behavioural paradigm (SST, n = 114; VIP, n = 152). **e**, Proportion of cells in CS– clusters for SST and VIP interneurons (SST, n = 78; VIP, n = 79). Chi-Square test ($\chi2(7) = 20.415$), $p = 0.0047$; SST vs. VIP, *post hoc* Chi-Square test with Bonferroni correction, 'Stable activated', $p = 0.0249$. **f**, Heatmap of CS+ responses in BLA interneurons across days (including data from all *GAD2-Cre*, *SST-Cre* and *VIP-Cre* mice) clustered into groups depending on their response pattern (n = 553 responsive cells; 'Tone activated'/activated before conditioning, n = 50; 'Tone inhibited'/inhibited before conditioning, n = 62; 'Fear'/activated after conditioning, n = 70; 'Fear inhibited'/inhibited after conditioning, n = 104; 'Extinction'/activated after extinction, n = 61; 'Extinction inhibited'/ inhibited after extinction, n = 50; 'Inhibited stable', n = 51; 'Activated stable', n = 105). **g**, Average traces of CS+ clusters shown in f. **h**, Heatmap of CS– responses in BLA interneurons across days (including data from all *GAD2-Cre*, *SST-Cre* and *VIP-Cre* mice) clustered into groups depending on their response pattern (n = 514 responsive cells; 'Tone activated'/activated before conditioning, n = 60; 'Tone inhibited'/inhibited before conditioning, n = 48; 'Fear'/activated after conditioning, n = 49; 'Fear inhibited'/inhibited after conditioning, n = 60; 'Extinction'/activated after extinction, n = 86; 'Extinction inhibited'/inhibited after extinction, n = 43; 'Inhibited stable', n = 63; 'Activated stable', n = 105). **i**, Average traces of CS– clusters shown in h.

Average traces in b, g and i are mean with s.e.m.; violin plots in c show distribution of all data points, Tukey box-and-whisker plots in show median values, 25th and 75th percentiles, and min to max whiskers with exception of outliers, dots indicate the mean. *$p < 0.05$, **$p < 0.01$. Additional details of statistical analyses are provided in Supplementary Table 1.

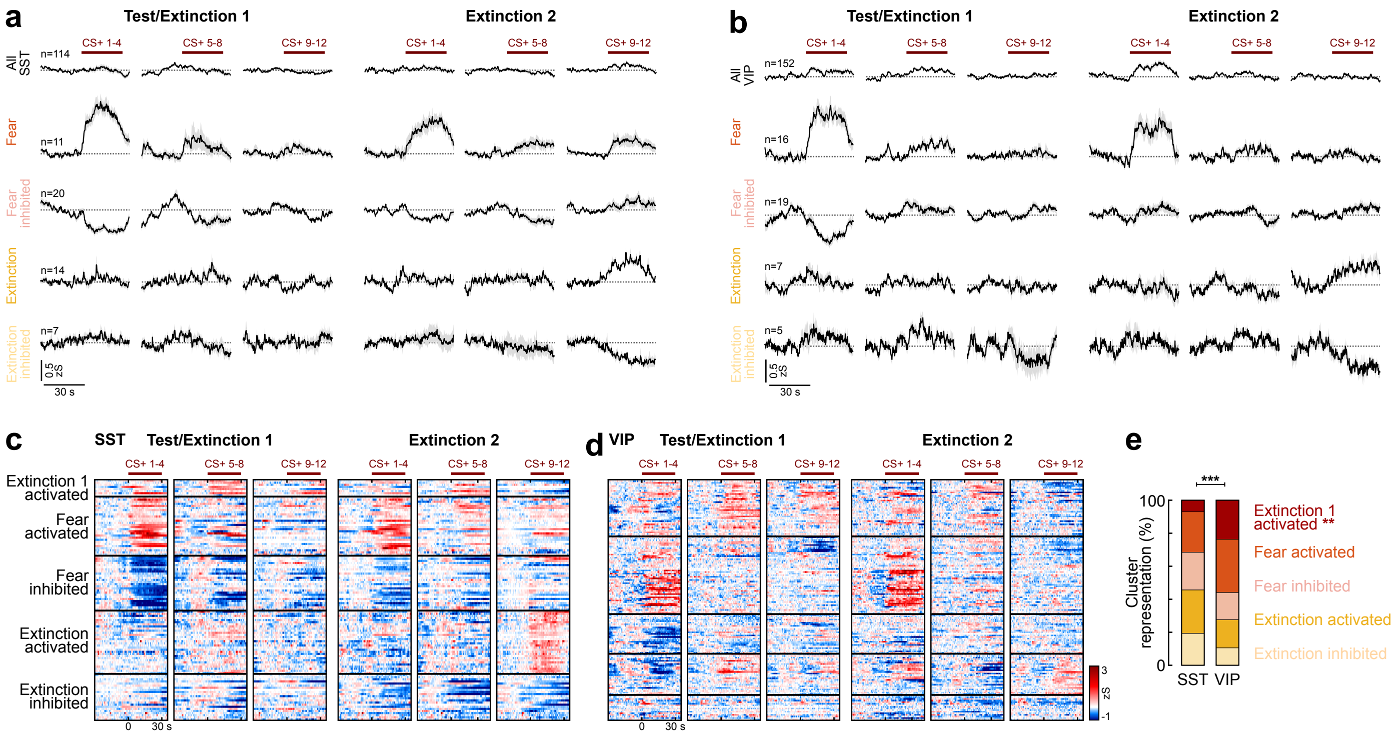

Supplementary Figure 13: Development of extinction responses in molecular interneuron subpopulations

a, Development of SST interneuron CS+ responses across extinction sessions separated by across-day clusters (see Figure 9). **b**, Development of VIP interneuron CS+ responses across extinction sessions separated by across-day clusters. **c**, Heatmap of CS+ responses in SST interneurons clustered into groups depending on their response pattern across the two extinction sessions (n = 114 SST interneurons; Extinction 1 activated, n = 8; Fear activated, n = 28; Fear inhibited, n = 26; Extinction activated, n = 30; Extinction inhibited, n = 22). **d**, Heatmap of CS+ responses in VIP interneurons clustered into groups depending on their response pattern across the two extinction sessions (n = 152 SST interneurons; Extinction 1 activated, n = 36; Fear activated, n = 49; Fear inhibited, n = 25; Extinction activated, n = 26; Extinction inhibited, n = 16). **e**, Corresponding proportion of cells in CS+ extinction clusters for SST and VIP interneurons (SST, n = 114; VIP, n = 152). Chi-Square test ($\chi2(4) = 19.773$), $p = 0.0006$; SST vs. VIP, *post hoc* Chi-Square test with Bonferroni correction, Extinction 1 activated, $p = 0.0028$.

Average traces in a and b are mean with s.e.m. **$p < 0.01$, ***$p < 0.001$. Additional details of statistical analyses are provided in Supplementary Table 1.

Supplementary References

75. Reéb, Z., Magyar, D., Weisz, F. *et al.* Morphological and electrophysiological diversity and connectivity of principal neurons in the lateral and basal nuclei of the mouse amygdala. *Sci. Rep.* **15**, 33675 (2025).

Supplementary Table 1: Summary of all statistical analyses for data presented in main and supplementary figures.

Main figures

Figure	Panel	Sample size	Statistical test	Individual comparisons
2	c	N = 9 mice	Friedman test<br>**χ2 = 11.53, *p* = 0.0016**	Dunn's multiple comparisons<br>CS+ vs. CS–, *p* > 0.9999<br>**CS+ vs. US, *p* = 0.0065**<br>**CS– vs. US, *p* = 0.0286**
2	e	N = 9 mice	Friedman test<br>**χ2 = 24.33, *p* < 0.0001**	Dunn's multiple comparisons<br>CS+/US vs. CS–/US, *p* > 0.9999<br>CS+/US vs. CS+/CS–, *p* = 0.1057<br>**CS+/US vs. CS+/CS–/US, *p* = 0.0004**<br>CS–/US vs. CS+/CS–, *p* = 0.0635<br>**CS–/US vs. CS+/CS–/US, *p* = 0.0002**<br>CS+/CS– vs. CS+/CS–/US, *p* = 0.6021
2	f	n = 519 cells	Chi-Square test<br>**χ2(2) = 6.3912, *p* = 0.0409**	Chi-Square with Bonferroni correction<br>(Activated vs. Inhibited)<br>**CS+, *p* < 0.0001**<br>CS–, *p* = 0.2027<br>US, *p* = 0.9177
2	h	n = 519 cells	–	Kolmogorov-Smirnov test with Bonferroni correction<br>CS+ vs. US, *p* = 1<br>CS– vs. US, *p* = 0.9668<br>CS+ vs. CS–, *p* = 1
3	e	n = 519 cells	Friedman test<br>χ2 = 1.598, *p* = 0.8091	–
3	f	n = 519 cells	Friedman test<br>χ2 = 2.402, *p* = 0.6623	–
3	h	N = 9 mice	Friedman test<br>**χ2 = 30.41, *p* < 0.0001**	Dunn's multiple comparisons<br>Activated stable vs. US off, *p* = 0.5314<br>Activated stable vs. Else, *p* = 0.0676<br>**Activated down vs. Activated up, *p* = 0.0327**<br>**Activated down vs. US off, *p* = 0.0023**<br>**Activated down vs. Else, *p* < 0.0001**<br>Inhibited stable vs. Else, *p* = 0.1854<br>Inhibited up vs. US off, *p* = 0.4609<br>Inhibited up vs. Else, *p* = 0.0566<br>All other comparisons, *p* > 0.9999
4	g	n = 519 cells	–	Paired Wilcoxon test with Bonferroni correction<br>(CS+ vs. CS–)<br>CS 1, *p* = 1<br>CS 2, *p* = 1<br>CS 3, *p* = 1<br>CS 4, *p* = 0.158<br>**CS 5, *p* = 0.0021**
4	h	CS+ n = 297 cells<br>CS– n = 312 cells	Chi-Square test<br>**χ2(6) = 117.19, *p* < 0.0001**	Chi-Square with Bonferroni correction (CS+ vs. CS–)<br>Up, *p* = 0.3987<br>Down inhibited, *p* = 0.2214<br>**Down activated, *p* < 0.0001**<br>**Stable, *p* < 0.0001**<br>First activated, *p* = 1<br>First inhibited, *p* = 1<br>Else, *p* = 0.0594
5	g	n = 519 cells	Before conditioning<br>Chi-Square test<br>χ2(2) = 2.4021, *p* = 0.3009	–
			After conditioning<br>Chi-Square test<br>χ2(2) = 0.1111, *p* = 0.9460	–
			After extinction<br>Chi-Square test<br>χ2(2) = 4.8741, *p* = 0.0874	–

5	h	CS+ n = 365 cells<br>CS– n = 357 cells	Chi-Square test<br>**χ2(7) = 105.84, *p* < 0.0001**	Chi-Square with Bonferroni correction (CS+ vs. CS–)<br>**Before conditioning activated, *p* = 0.0275**<br>Before conditioning inhibited, *p* = 1<br>**After conditioning activated, *p* = 0.0016**<br>After conditioning inhibited, *p* = 0.2213<br>**After extinction activated, *p* = 0.0074**<br>After extinction inhibited, *p* = 0.5894<br>Stable inhibited, *p* = 1<br>**Stable activated, *p* < 0.0001**
6	c	Fear n = 66<br>Fear inhibited n = 48<br>Extinction n = 63<br>Extinction inhibited n = 33	Start<br>Kruskal Wallis test<br>***H* = 10.83, *p* = 0.0127**	**Fear vs. Fear inhibited, *p* = 0.0173**<br>Fear vs. Extinction, *p* = 0.2240<br>Fear vs. Extinction inhibited, *p* > 0.9999<br>Fear inhibited vs. Extinction, *p* > 0.9999<br>Fear inhibited vs. Extinction inhibited, *p* = 0.1700<br>Extinction vs. Extinction inhibited, *p* = 0.9981
			Stop<br>Kruskal Wallis test<br>***H* = 19.86, *p* = 0.0002**	**Fear vs. Fear inhibited, *p* = 0.0015**<br>Fear vs. Extinction, *p* = 0.1351<br>Fear vs. Extinction inhibited, *p* > 0.9999<br>Fear inhibited vs. Extinction, *p* = 0.7679<br>**Fear inhibited vs. Extinction inhibited, *p* = 0.0015**<br>Extinction vs. Extinction inhibited, *p* = 0.0762
7	d	N = 9 mice	Friedman test<br>χ2 = 5, *p* = 0.172	–
7	e	N = 9 mice	CS+<br>Friedman test<br>**χ2 = 13.9, *p* = 0.0030**	Dunn's multiple comparisons<br>**Pairing 2 vs. Pairing 3, *p* = 0.0115**<br>**Pairing 2 vs. Pairing 4, *p* = 0.0209**<br>**Pairing 2 vs. Pairing 5, *p* = 0.0115**<br>Pairing 3 vs. Pairing 4, *p* > 0.9999<br>Pairing 3 vs. Pairing 5, *p* > 0.9999<br>Pairing 4 vs. Pairing 5, *p* > 0.9999
			CS–<br>Friedman test<br>**χ2 = 14.2, *p* = 0.0026**	Dunn's multiple comparisons<br>**Pairing 2 vs. Pairing 3, *p* = 0.0370**<br>**Pairing 2 vs. Pairing 4, *p* = 0.0115**<br>**Pairing 2 vs. Pairing 5, *p* = 0.0061**<br>Pairing 3 vs. Pairing 4, *p* > 0.9999<br>Pairing 3 vs. Pairing 5, *p* > 0.9999<br>Pairing 4 vs. Pairing 5, *p* > 0.9999
7	f	N = 9 mice	Friedman test<br>**χ2 = 13.9, *p* = 0.0304**	Dunn's multiple comparisons<br>All vs. Up, *p* = 0.4309<br>All vs. Stable, *p* > 0.9999<br>All vs. Down inhibited, *p* > 0.9999<br>All vs. First activated, *p* > 0.9999<br>All vs. First inhibited, *p* > 0.9999<br>All vs. Else, *p* > 0.9999
7	g	N = 9 mice	Friedman test<br>**χ2 = 36.0, *p* < 0.0001**	Dunn's multiple comparisons<br>**All vs. Activated stable, *p* = 0.0272**<br>All vs. Activated down, *p* = 0.2699<br>All vs. Activated up, *p* = 0.0754<br>All vs. Inhibited stable, *p* > 0.9999<br>All vs. Inhibited up, *p* > 0.9999<br>All vs. US off, *p* = 0.9508<br>All vs. Else, *p* > 0.9999
7	h	N = 9 mice	CS+<br>Friedman test<br>χ2 = 2.89, *p* = 0.2781	–
			CS–<br>Friedman test<br>**χ2 = 14.2, *p* < 0.0001**	Dunn's multiple comparisons<br>**Conditioning vs. After conditioning, *p* = 0.0005**<br>Conditioning vs. After extinction, *p* = 0.1780<br>After conditioning vs. After extinction, *p* = 0.1780
8	c	SST n = 114 cells<br>VIP n = 101 cells	–	Mann-Whitney test with Bonferroni correction (SST vs. VIP)<br>**US 1, *p* < 0.0001**<br>**US 2, *p* < 0.0001**<br>**US 3, *p* < 0.0001**<br>US 4, *p* = 0.6850<br>**US 5, *p* = 0.0005**

8	f	SST n = 114 cells<br>VIP n = 101 cells	–	Mann-Whitney test with Bonferroni correction<br>(SST vs. VIP)<br>**CS+ 1, *p* < 0.0001**<br>CS+ 2, *p* = 1<br>**CS+ 3, *p* < 0.0001**<br>**CS+ 4, *p* = 0.0287**<br>**CS+ 5, *p* = 0.0003**
8	g	SST n = 114 cells<br>VIP n = 101 cells	CS+<br>Chi-Square test<br>**χ2(2) = 30.885, *p* < 0.0001**	Chi-Square with Bonferroni correction (SST vs. VIP)<br>**Activated, *p* < 0.0001**<br>**Inhibited, *p* = 0.0004**<br>No response, *p* = 0.2852
			CS–<br>Chi-Square test<br>χ2(2) = 2.5014, *p* = 0.2863	–
			US<br>Chi-Square test<br>**χ2(2) = 16.663, *p* = 0.0002**	Chi-Square with Bonferroni correction (SST vs. VIP)<br>**Activated, *p* = 0.0028**<br>**Inhibited, *p* = 0.0007**<br>No response, *p* = 1
8	h	SST n = 89 cells<br>VIP n = 80 cells	Chi-Square test<br>**χ2(6) = 28.635, *p* < 0.0001**	Chi-Square with Bonferroni correction (SST vs. VIP)<br>**Activated stable, *p* = 0.0001**<br>Activated down, *p* = 1<br>Activated up, *p* = 0.6272<br>Inhibited stable, *p* = 0.0978<br>Inhibited up, *p* = 0.7564<br>US off, *p* = 1<br>Else, *p* = 1
8	i	SST n = 63 cells<br>VIP n = 68 cells	Chi-Square test<br>**χ2(5) = 28.155, *p* < 0.0001**	Chi-Square with Bonferroni correction (SST vs. VIP)<br>Up, *p* = 0.3152<br>Down inhibited, *p* = 0.7037<br>**Stable activated, *p* = 0.0046**<br>First activated, *p* = 1<br>**First inhibited, *p* = 0.0418**<br>Else, *p* = 0.1501
8	j	SST n = 74 cells<br>VIP n = 55 cells	Chi-Square test<br>χ2(5) = 9.5089, *p* = 0.0904	–
9	c	SST n = 114 cells<br>VIP n = 152 cells	SST<br>–	Paired Wilcoxon test with Bonferroni correction<br>Before conditioning vs. After conditioning, *p* = 1<br>**Before conditioning vs. After extinction, *p* = 0.036**<br>After conditioning vs. After extinction, *p* = 0.780
			VIP<br>–	Paired Wilcoxon test with Bonferroni correction<br>Before conditioning vs. After conditioning, *p* = 1<br>**Before conditioning vs. After extinction, *p* = 0.012**<br>After conditioning vs. After extinction, *p* = 0.216
9	d	SST n = 114 cells<br>VIP n = 152 cells	Before conditioning<br>Chi-Square test<br>**χ2(2) = 9.7873, *p* = 0.0075**	Chi-Square with Bonferroni correction (SST vs. VIP)<br>Activated, *p* = 1<br>**Inhibited, *p* = 0.0098**<br>No response, *p* = 0.3386
			After conditioning<br>Chi-Square test<br>**χ2(2) = 6.5609, *p* = 0.0376**	Chi-Square with Bonferroni correction (SST vs. VIP)<br>Activated, *p* = 1<br>**Inhibited, *p* = 0.0496**<br>No response, *p* = 0.4349
			After extinction<br>Chi-Square test<br>**χ2(2) =23.938, *p* < 0.0001**	Chi-Square with Bonferroni correction (SST vs. VIP)<br>**Activated, *p* = 0.0005**<br>Inhibited, *p* = 0.1987<br>**No response, *p* < 0.0001**
9	e	SST n = 92 cells<br>VIP n = 96 cells	Chi-Square test<br>χ2(7) = 13.065, *p* = 0.0705	–
9	h	SST n = 114 cells<br>VIP n = 152 cells	Kruskal Wallis test<br>***H* = 15.33, *p* = 0.0016**	**SST start vs. SST stop, *p* = 0.0025**<br>VIP start vs. VIP stop, *p* > 0.9999<br>SST start vs. VIP start, *p* > 0.9999<br>**SST stop vs. VIP stop, *p* = 0.0177**

Supplementary Figures

Figure	Panel	Sample size	Statistical test	Individual comparisons
S3	b	N = 9 mice	Friedman test<br>**χ2 = 11.26, p = 0.0465**	Dunn's multiple comparisons<br>Down inhibited vs. Stable, p = 0.1762<br>Stable vs. First inhibited, p = 0.6573<br>Stable vs. Else, p = 0.0836<br>All other comparisons, p > 0.9999
S3	c	N = 9 mice	Friedman test<br>χ2 = 7.216, p = 0.2050	–
S5	e	CS+ n = 165 cells<br>CS– n = 185 cells	Chi-Square test<br>χ2(1) = 0.5135, p = 0.4736	–
S5	f	N = 9 mice	–	Paired Wilcoxon test p = 0.1289
S5	g	N = 9 mice	–	Paired Wilcoxon test p = 0.7734
S5	l	12 kHz n = 202 cells<br>6 kHz n = 148 cells	Chi-Square test<br>χ2(1) = 0.5361, p = 0.4641	–
S6	d	n = 519 cells	–	Paired Wilcoxon test with Bonferroni correction<br>(CS+ vs. CS–)<br>Before conditioning, p = 0.169<br>After conditioning, p = 1<br>After extinction, p = 1
S6	e	N = 9 mice	Friedman test<br>χ2 = 6.299, p = 0.3905	–
S6	f	N = 9 mice	Friedman test<br>**χ2 = 25.22, p = 0.0007**	Dunn's multiple comparisons<br>Tone activated vs. Tone inhibited, p = 0.6648<br>**Tone activated vs. Fear, p = 0.0494**<br>**Tone activated vs. Fear inhibited, p = 0.0072**<br>**Tone activated vs. Extinction, p = 0.0212**<br>**Tone activated vs. Extinction inhibited, p = 0.0022**<br>Tone activated vs. Inhibited stable, p = 0.5151<br>Fear inhibited vs. Activated stable, p = 0.5151<br>Extinction inhibited vs. Activated stable, p = 0.2279<br>All other comparisons, p > 0.9999
S7	f	Fear n = 66<br>Fear inhibited n = 48<br>Extinction n = 63<br>Extinction inhibited<br>n = 33	Start<br>Kruskal Wallis test<br>**H = 9.836, p = 0.0200**	Fear vs. Fear inhibited, p = 0.1226<br>Fear vs. Extinction, p > 0.9999<br>Fear vs. Extinction inhibited, p > 0.9999<br>Fear inhibited vs. Extinction, p > 0.9999<br>**Fear inhibited vs. Extinction inhibited, p = 0.0255**<br>Extinction vs. Extinction inhibited, p = 0.3249
			Stop<br>Kruskal Wallis test<br>**H = 14.83, p = 0.0020**	**Fear vs. Fear inhibited, p = 0.0057**<br>Fear vs. Extinction, p > 0.9999<br>Fear vs. Extinction inhibited, p > 0.9999<br>Fear inhibited vs. Extinction, p = 0.2440<br>**Fear inhibited vs. Extinction inhibited, p = 0.0058**<br>Extinction vs. Extinction inhibited, p = 0.5983
S7	h	Fear n = 66<br>Fear inhibited n = 48<br>Extinction n = 63<br>Extinction inhibited<br>n = 33	Start<br>Kruskal Wallis test<br>**H = 10.89, p = 0.0123**	**Fear vs. Fear inhibited, p = 0.0095**<br>Fear vs. Extinction, p = 0.1357<br>Fear vs. Extinction inhibited, p = 0.7794<br>Fear inhibited vs. Extinction, p > 0.9999<br>Fear inhibited vs. Extinction inhibited, p > 0.9999<br>Extinction vs. Extinction inhibited, p > 0.9999
			Stop<br>Kruskal Wallis test<br>**H = 14.19, p = 0.0027**	**Fear vs. Fear inhibited, p = 0.0135**<br>Fear vs. Extinction, p = 0.2639<br>Fear vs. Extinction inhibited, p > 0.9999<br>Fear inhibited vs. Extinction, p > 0.9999<br>**Fear inhibited vs. Extinction inhibited, p = 0.0132**<br>Extinction vs. Extinction inhibited, p = 0.1776
S8	c	N = 9 mice	Friedman test<br>χ2 = 1.933, p = 0.5864	–

S10	e	N = 4 mice	Friedman test<br>**χ2 = 6.500, *p* = 0.0417**	Dunn's multiple comparisons<br>CS+ vs. CS–, *p* > 0.9999<br>**CS+ vs. US, *p* = 0.0400**<br>CS– vs. US, *p* = 0.2321
S10	f	N = 4 mice	Friedman test<br>**χ2 = 9.711, *p* = 0.0058**	Dunn's multiple comparisons<br>CS+/US vs. CS–/US, *p* > 0.9999<br>CS+/US vs. CS+/CS–, *p* > 0.9999<br>CS+/US vs. CS+/CS–/US, *p* = 0.0557<br>CS–/US vs. CS+/CS–, *p* > 0.9999<br>CS–/US vs. CS+/CS–/US, *p* = 0.0557<br>CS+/CS– vs. CS+/CS–/US, *p* > 0.9999
S10	g	N = 4 mice	Friedman test<br>χ2 = 4.133, *p* = 0.1574	–
S10	h	N = 4 mice	Friedman test<br>**χ2 = 8.595, *p* = 0.0168**	Dunn's multiple comparisons<br>CS+/US vs. CS–/US, *p* > 0.9999<br>CS+/US vs. CS+/CS–, *p* > 0.9999<br>**CS+/US vs. CS+/CS–/US, *p* = 0.0370**<br>CS–/US vs. CS+/CS–, *p* > 0.9999<br>CS–/US vs. CS+/CS–/US, *p* = 0.3314<br>CS+/CS– vs. CS+/CS–/US, *p* > 0.9999
S11	g	SST n = 114 cells<br>VIP n = 101 cells	–	Mann-Whitney test with Bonferroni correction<br>(SST vs. VIP)<br>CS– 1, *p* = 1<br>CS– 2, *p* = 1<br>CS– 3, *p* = 1<br>CS– 4, *p* = 1<br>CS– 5, *p* = 1
S12	c	SST n = 114 cells<br>VIP n = 152 cells	SST<br>–	Paired Wilcoxon test with Bonferroni correction<br>Before conditioning vs. After conditioning, *p* = 0.222<br>Before conditioning vs. After extinction, *p* = 1<br>After conditioning vs. After extinction, *p* = 0.828
			VIP<br>–	Paired Wilcoxon test with Bonferroni correction<br>Before conditioning vs. After conditioning, *p* = 1<br>Before conditioning vs. After extinction, *p* = 0.384<br>After conditioning vs. After extinction, *p* = 1
S12	d	SST n = 114 cells<br>VIP n = 152 cells	Before conditioning<br>Chi-Square test<br>χ2(2) = 0.6568, *p* = 0.7201	–
			After conditioning<br>Chi-Square test<br>χ2(2) = 4.9771, *p* = 0.0830	–
			After extinction<br>Chi-Square test<br>χ2(2) = 5.1876, *p* = 0.0747	–
S12	e	SST n = 78 cells<br>VIP n = 79 cells	Chi-Square test<br>**χ2(7) = 20.415, *p* = 0.0047**	Chi-Square with Bonferroni correction (SST vs. VIP)<br>Before conditioning activated, *p* = 1<br>Before conditioning inhibited, *p* = 1<br>After conditioning activated, *p* = 1<br>After conditioning inhibited, *p* = 1<br>After extinction activated, *p* = 0.0594<br>After extinction inhibited, *p* = 1<br>Stable inhibited, *p* = 1<br>**Stable activated, *p* = 0.0249**
S13	e	SST n = 114 cells<br>VIP n = 152 cells	Chi-Square test<br>**χ2(4) = 19.773, *p* = 0.0006**	Chi-Square with Bonferroni correction (SST vs. VIP)<br>**Extinction 1 activated, *p* = 0.0028**<br>Fear activated, *p* = 1<br>Fear inhibited, *p* = 1<br>Extinction activated, *p* = 0.4731<br>Extinction inhibited, *p* = 0.3243
