# Supplementary material for: Heterogeneous plasticity of amygdala interneurons in associative learning and extinction
Source: Nat Commun. 2025 Nov 11;16:9926. doi: 10.1038/s41467-025-66122-y (PMC12614800; doi:10.1038/s41467-025-66122-y)
Supplement: Supplementary file 2 — Description of Additional Supplementary Files [file 41467_2025_66122_MOESM2_ESM.pdf]

Description of Additional Supplementary Files

Supplementary Movie 1. Example video of fear conditioning session showing interneuron activity during first and last CS+ (30s) - US (2s) pairing (speed 2x).
