# Supplementary material for: Heterogeneous plasticity of amygdala interneurons in associative learning and extinction
Source: Nat Commun. 2025 Nov 11;16:9926. doi: 10.1038/s41467-025-66122-y (PMC12614800; doi:10.1038/s41467-025-66122-y)
Supplement: Supplementary file 4 — Reporting Summary [file 41467_2025_66122_MOESM4_ESM.pdf]

nature portfolio

Corresponding author(s): Sabine Krabbe

Last updated by author(s): Oct 19, 2025

Reporting Summary

Nature Portfolio wishes to improve the reproducibility of the work that we publish. This form provides structure for consistency and transparency in reporting. For further information on Nature Portfolio policies, see our Editorial Policies and the Editorial Policy Checklist.

Statistics

For all statistical analyses, confirm that the following items are present in the figure legend, table legend, main text, or Methods section.

n/a	Confirmed	
[ ]	[x]	The exact sample size (*n*) for each experimental group/condition, given as a discrete number and unit of measurement
[ ]	[x]	A statement on whether measurements were taken from distinct samples or whether the same sample was measured repeatedly
[ ]	[x]	The statistical test(s) used AND whether they are one- or two-sided<br>*Only common tests should be described solely by name; describe more complex techniques in the Methods section.*
[ ]	[x]	A description of all covariates tested
[ ]	[x]	A description of any assumptions or corrections, such as tests of normality and adjustment for multiple comparisons
[ ]	[x]	A full description of the statistical parameters including central tendency (e.g. means) or other basic estimates (e.g. regression coefficient) AND variation (e.g. standard deviation) or associated estimates of uncertainty (e.g. confidence intervals)
[ ]	[x]	For null hypothesis testing, the test statistic (e.g. *F*, *t*, *r*) with confidence intervals, effect sizes, degrees of freedom and *P* value noted<br>*Give P values as exact values whenever suitable.*
[x]	[ ]	For Bayesian analysis, information on the choice of priors and Markov chain Monte Carlo settings
[x]	[ ]	For hierarchical and complex designs, identification of the appropriate level for tests and full reporting of outcomes
[ ]	[x]	Estimates of effect sizes (e.g. Cohen's *d*, Pearson's *r*), indicating how they were calculated

Our web collection on statistics for biologists contains articles on many of the points above.

Software and code

Policy information about availability of computer code

Data collection	Zeiss ZEN Black 2010 (Carl Zeiss AG)<br>VisiView5.0 (Visitron Systems GmbH)<br>nVista HD 2.1 software (Inscopix)<br>RPvdsEx 78 software (Tucker-Davis Technologies)<br>Radiant 2.0 software (Plexon)<br>MAP 2.7 system (Plexon)<br>CinePlex Studio 3.4.1 software (Plexon)
Data analysis	MATLAB R2021b, R 4.1.0, RStudio 2023.12.1, Python 3.10.8<br>Python packages: caiman 1.9.13, plotly 5.15.0, pandas 1.2.23<br>R packages: rstatix 0.7.2, tidyverse 1.3.2<br>MATLAB packages: CIAtah 4.5.9<br>Special MATLAB functions: fitcsv, fitcecoc, trapz, evalclusters, crossval<br>ImageJ 2.0.0-rc-49/1.51a (NIH)<br>Prism 10 (GraphPad Software)<br>Imaris 9.9.1 (Bitplane)

For manuscripts utilizing custom algorithms or software that are central to the research but not yet described in published literature, software must be made available to editors and reviewers. We strongly encourage code deposition in a community repository (e.g. GitHub). See the Nature Portfolio guidelines for submitting code & software for further information.

Data

Policy information about availability of data

All manuscripts must include a data availability statement. This statement should provide the following information, where applicable:

- Accession codes, unique identifiers, or web links for publicly available datasets
- A description of any restrictions on data availability
- For clinical datasets or third party data, please ensure that the statement adheres to our policy

The source data underlying main figures and supplementary information are available as Source Data file. Custom code and full datasets are available on Zenodo (data DOI 10.5281/zenodo.17390680; code DOI 10.5281/zenodo.17390682).

Research involving human participants, their data, or biological material

Policy information about studies with human participants or human data. See also policy information about sex, gender (identity/presentation), and sexual orientation and race, ethnicity and racism.

Reporting on sex and gender	not applicable
Reporting on race, ethnicity, or other socially relevant groupings	not applicable
Population characteristics	not applicable
Recruitment	not applicable
Ethics oversight	not applicable

Note that full information on the approval of the study protocol must also be provided in the manuscript.

Field-specific reporting

Please select the one below that is the best fit for your research. If you are not sure, read the appropriate sections before making your selection.

- [x] Life sciences
- [ ] Behavioural & social sciences
- [ ] Ecological, evolutionary & environmental sciences

For a reference copy of the document with all sections, see nature.com/documents/nr-reporting-summary-flat.pdf

Life sciences study design

All studies must disclose on these points even when the disclosure is negative.

Sample size	No statistical methods were used to predetermine sample size. The sample sizes were chosen based on published studies in the field.
Data exclusions	No data was excluded from the analysis, with the exception of N=2 VIP-Cre mice: data from these animals were excluded from the conditioning day analysis as one of the five US exposures could not be verified with the behavioural recordings. However, these mice were included for across-day analysis, since they learned the association between CS+ and US with the remaining 4 pairings.
Replication	The number of repetitions are indicated in figure legends and supplementary tables. All findings were replicable within groups.
Randomization	No formal randomisation was used. Animals from different groups were subjected to behavioural paradigms in mixed cohorts. Frequencies for CS+ and CS– were counterbalanced across mice.
Blinding	Blinding was not performed per se as data across samples were pooled for analysis and later separated for cell type.

Reporting for specific materials, systems and methods

We require information from authors about some types of materials, experimental systems and methods used in many studies. Here, indicate whether each material, system or method listed is relevant to your study. If you are not sure if a list item applies to your research, read the appropriate section before selecting a response.

Materials & experimental systems

n/a	Involved in the study	
☐	☒	Antibodies
☒	☐	Eukaryotic cell lines
☒	☐	Palaeontology and archaeology
☐	☒	Animals and other organisms
☒	☐	Clinical data
☒	☐	Dual use research of concern
☒	☐	Plants

Methods

n/a	Involved in the study	
☒	☐	ChIP-seq
☒	☐	Flow cytometry
☒	☐	MRI-based neuroimaging

Antibodies

Antibodies used

primary antibodies:
rabbit anti-VIP (Immunostar, 20077, LOT# 1339001, 1:1000)
guinea pig anti-VIP (Synaptic Systems, 443005, LOT# 3-11, 1:500)
rat anti-SST (Merck Millipore, MAB354, LOT# 232625 and 3474070, 1:500)
guinea pig anti-PV (Synaptic Systems, 195004, LOT# 195004/10, 1:500)
guinea pig anti-PV (Synaptic Systems, 195308, LOT# 1-3, 1-9, 1:500)
rabbit anti-pro-CCK (Frontiers Institute, CCK-pro-Rb-Af350, LOT# 453, 1:500)
chicken anti GFP (Thermo Fisher Scientific, A10262, LOT# 2738236, 1:1000)

secondary antibodies:
goat anti-rabbit Alexa Fluor 647 (Thermo Fisher Scientific, A21245, Lot# 1778005, 1:750)
goat anti-rat Alexa Fluor 568 (Thermo Fisher Scientific, A11077, Lot# 692966 and 2217022, 1:750)
goat anti-guinea pig DyLight 405 (Jackson ImmunoResearch, 106-475-003, Lot# 126016, 1:250)
goat anti-chicken Alexa Fluor 488 (Thermo Fisher Scientific, A11039, Lot# 2420700, 1:750)
goat anti-rabbit Alexa Fluor 568 (Thermo Fisher Scientific, A11011, Lot# 2782620, 1:750)
goat anti-guinea pig Alexa Fluor 647 (Thermo Fisher Scientific, A21450, Lot# 2231672, 1:750)

Protocols are indicated in the respective Methods section.

Validation

All antibodies are widely used commercially available antibodies. Antibodies were validated in WB and IHC by the manufacturer. Specificity of secondary antibodies was further validated by omitting preceding primary antibody incubation.

Animals and other research organisms

Policy information about studies involving animals; ARRIVE guidelines recommended for reporting animal research, and Sex and Gender in Research

Laboratory animals

Mus musculus, transgenic lines:
GAD2-Ires-Cre (Jackson Laboratory #028867)
VIP-Ires-Cre (Jackson Laboratory #010908)
SST-Ires-Cre (Jackson Laboratory #028864)

Only heterozygous mice were used. Mouse lines were fully backcrossed to a C57BL/6J background (Jackson Laboratory #000664). Animals were kept in a 12 h light/dark cycle (22-24 °C, 40-60% humidity) with access to food and water ad libitum.

Wild animals

not applicable

Reporting on sex

Experiments were performed with male (GAD2-Cre, VIP-Cre, SST-Cre) and female (GAD2-Cre) mice aged 2-3 months at the time of injection. No post hoc sex- or gender-based analysis was performed due to low sample size.

Field-collected samples

not applicable

Ethics oversight

All animal procedures were performed in accordance with institutional guidelines at the Friedrich Miescher Institute for Biomedical Research, and were approved by the Veterinary Department of the Canton of Basel-Stadt before starting experiments.

Note that full information on the approval of the study protocol must also be provided in the manuscript.

Plants

Seed stocks

Report on the source of all seed stocks or other plant material used. If applicable, state the seed stock centre and catalogue number. If plant specimens were collected from the field, describe the collection location, date and sampling procedures.

Novel plant genotypes

Describe the methods by which all novel plant genotypes were produced. This includes those generated by transgenic approaches, gene editing, chemical/radiation-based mutagenesis and hybridization. For transgenic lines, describe the transformation method, the number of independent lines analyzed and the generation upon which experiments were performed. For gene-edited lines, describe the editor used, the endogenous sequence targeted for editing, the targeting guide RNA sequence (if applicable) and how the editor was applied.

Authentication

Describe any authentication procedures for each seed stock used or novel genotype generated. Describe any experiments used to assess the effect of a mutation and, where applicable, how potential secondary effects (e.g. second site T-DNA insertions, mosiacism, off-target gene editing) were examined.
